# Developing Strategies to Help Bee Colony Resilience in Changing Environments

**DOI:** 10.3390/ani12233396

**Published:** 2022-12-02

**Authors:** Isabelle Dequenne, Jean-Michel Philippart de Foy, Patrice D. Cani

**Affiliations:** 1 J-M Philippart de Foy & I Dequenne Consultation, Avenue Orban, 127, 1150 Brussels, Belgium; 2 Metabolism and Nutrition Research Group, Louvain Drug Research Institute, UCLouvain, Université Catholique de Louvain, 1200 Brussels, Belgium; 3 WELBIO Department, WEL Research Institute, Walloon Excellence in Life Sciences and BIOtechnology (WELBIO), Avenue Pasteur, 6, 1300 Wavre, Belgium

**Keywords:** honeybee health, nutrition, microbiota strategies, resilience

## Abstract

**Simple Summary:**

Modifications to natural environments demand that high-level adaptations be made by living beings in order to continue thriving in their specific ecosystems. Multiple scientific studies have unraveled the strengths and weaknesses of honeybees and may guide efforts of the beekeeping world in developing strategies favoring their resilience and survival. These strategies include nutrition and microbiota management, or parasite elimination and pesticide detoxification.

**Abstract:**

Climate change, loss of plant biodiversity, burdens caused by new pathogens, predators, and toxins due to human disturbance and activity are significant causes of the loss of bee colonies and wild bees. The aim of this review is to highlight some possible strategies that could help develop bee resilience in facing their changing environments. Scientists underline the importance of the links between nutrition, microbiota, and immune and neuroendocrine stress resistance of bees. Nutrition with special care for plant-derived molecules may play a major role in bee colony health. Studies have highlighted the importance of pollen, essential oils, plant resins, and leaves or fungi as sources of fundamental nutrients for the development and longevity of a honeybee colony. The microbiota is also considered as a key factor in bee physiology and a cornerstone between nutrition, metabolism, growth, health, and pathogen resistance. Another stressor is the varroa mite parasite. This parasite is a major concern for beekeepers and needs specific strategies to reduce its severe impact on honeybees. Here we discuss how helping bees to thrive, especially through changing environments, is of great concern for beekeepers and scientists.

## 1. Introduction

**Figure 1:**
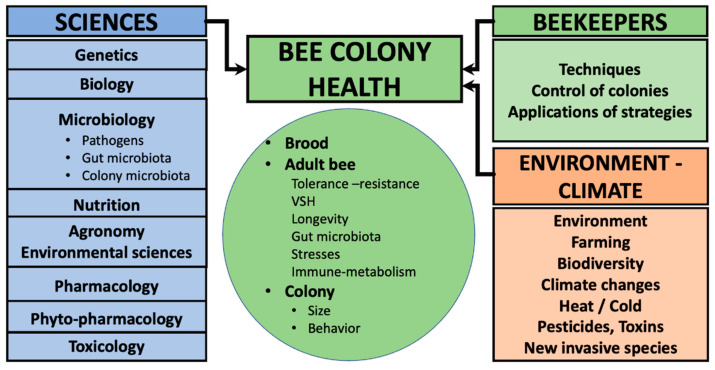
Matrix of a transdisciplinary system. Bee health is a Systems Medicine. Future research centered on bee health should involve integrating fundamental science with agronomy and environmental sciences, and beekeepers and farmers in a collaborative way in order to face the changing environment.

The high level of annual losses of honeybee colonies over the past few decades is of great concern for beekeeping. Indeed, bee health is linked to human health [1,2,3,4,5,6,7] and unravelling the complex interactions between the honeybee colonies and their environment requires numerous tools and continued progress in epigenetics and nutrigenomics in order to identify stress factors endangering honeybees (Figure 1) [8,9,10,11,12,13,14,15]. In this review, we are not performing an intensive study of all the strategies for bee colony survival and health, but rather, we are gathering the current knowledge on how specific interventions and nutritional approaches might contribute to sustaining their resilience. Although, there is no simple answer, it is important to dig into the potential complexity and interrelationship of biological systems and environmental factors [16]. Indeed, scientists suggest multifactorial interconnected weblike causes and effects on the relationship between a beehive and its environment (Figure 1), as recently reviewed by Knoll et al. investigating seasonal transition to winter and how honeybee colonies can survive during seasonal adaptation [16]. Nowadays, epigenetic and nutrigenomic studies show the importance of interactions between nutrition (diversity and quality), microbiota, climate, toxins, pathogens, and the resilience of a honeybee colony facing environmental changes (Figure 1) [8,11,17,18,19,20,21,22,23,24,25,26,27,28,29,30,31,32,33,34,35,36,37,38]. Among the key factors, data show specific relations between bee immunosuppression and the combined effects of malnutrition (we still have to define what is adequate nutrition), parasites, microbial pathogens (bacteria and/or viruses), pesticide exposure from agriculture or even beekeeping, poor environments, human-modified environments, monofloral intensive agriculture, and stress linked to cold or drought [14,22,24,39].

## 2. Bees Are Superorganisms: Challenges and Strategies to Optimize Their Health

**Figure 2:**
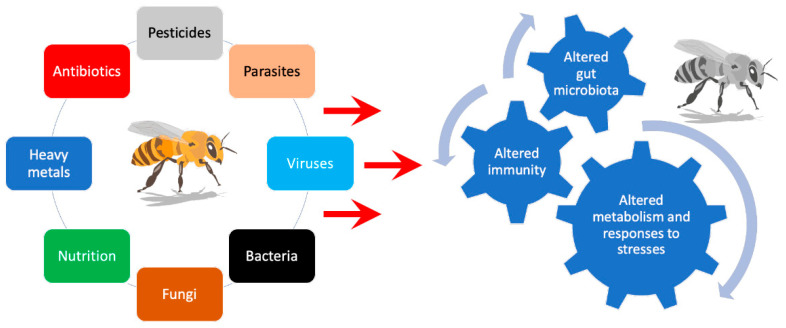
Factors affecting a bee’s metabolism and responses to stress.

The scientific community now considers a bee colony as a superorganism with integrated interacting individuals, adapting to the environment from an individual level of immunology and metabolism to colony-based behavior and responses [12,30,40,41]. Hence, developing strategies to help bees adapt to stress starts by understanding what stress is about [16]. Stress is about adaptation to a challenge activating the individual’s or colony’s homeostasis, as well as activating neuroendocrine, metabolic and immune mechanisms, and social organization. Many stress factors have been proposed and can be physical, chemical, nutritional, microbiological, and psychosocial. Stress may be generated by the perception of danger, a pathogen or parasite attack, nutrient deficiency, malnutrition, metabolic imbalance, microbiota disruption, or dysfunctional metabolic homeostasis induced by physical challenges: climate change, heat, cold, drought, and toxins (e.g., pesticides, antibiotics, heavy metals, air pollution, nanoparticles, plastics) (Figure 2) [9,11,13,14,16,17,18,19,20,21,22,24,27,28,29,30,39,42,43,44,45,46,47,48,49,50,51,52,53,54,55,56]. Based on the current evidence showing that honeybees and colonies are under the influence of numerous factors, a key question is: Could unravelling all those complex interactions allow the scientific community to set strategies that would help beekeepers reduce the loss of colonies ? The answer to this question requires a specific awareness from stakeholders in view of sharing and developing concrete plans proposed by both national and worldwide politics.

There is no “one” strategy. To understand part of the basic elements, we must consider the roles that nutrition and the microbiota (both having an impact on the immune and neuroendocrine system), anti-parasite techniques, and adaptations to new invasive species (*Vespa velutina nigrithorax*, for instance) all playing in bee health and resilience.

## 3. The Role of Nutrition

In this section of the review, we are addressing specific points related to nutrition. Key questions will be debated such as: What benefit will bees get from quality feeding for winter? Why do we have to care about the quality and diversity of nutrition for our honeybees?

Malnutrition is a major factor impacting animal health, resistance to pathogens, gut microbiota, general health, and survival [3]. It is of utmost importance to understand the complex interactions between disease, external stressors from the environment, and threats within the colony that can impact the health and survival of honeybees [16]. Nowadays, it is commonly accepted that several stressors might be considered, including external, anthropologic, or natural stressors. Among these, we can cite exposure to pesticides from agriculture or even beekeeping activities, the loss of floral biodiversity on farmlands, seasonal changes, climate changes, and any environmental stress. In addition, external stressors and some internal stressors have been described that can affect the general survival of a colony [13,16,24,37,57]. The most common factors are parasites (*Varroa destructor*), microbial pathogens (*Paenibacillus larvae*, *Melissococcus plutonius*), malnutrition, and genetics (Figure 2).

Some authors have summarized these factors as the heuristic 4P, which stands for Poor environment and nutrition, Parasites, Pathogens, and Pesticides [14,58]. Nutritional quality and diversity are considered as crucial factors for supporting individual and colony health [3,4,5,6,7]. It is generally admitted that all these elements together (the multifactorial effect) contribute to and induce bee immunosuppression leading to colony weakening and losses seen worldwide. According to the literature published up to now, the biggest challenge for honeybees comes from immunosuppression and microbiota alteration induced by the combined effects of *Varroa destructor*, viruses, pesticides, and malnutrition (Figure 1 and Figure 2).

Over the last two decades, the microbiota has been largely studied in the context of human health and is linked with numerous pathological situations [8,11,17,22,23,24,28,32,33,35,43,48,50,59,60,61,62,63,64,65,66,67,68,69,70,71,72,73,74,75,76,77,78,79,80,81,82,83,84,85,86,87]. Interestingly, the microbiota is also seen as a cornerstone to honeybee health, as for so many other living organisms. Gut microbes help in food digestion, and have adapted to long, co-evolutionary selection. They play a major role in host metabolism, immune stimulation, neuroendocrine regulation, longevity, and global health. They also help in pesticide detoxification and resistance against pathogen invasion [8,24,31,32,33,36,37,48,57,63,86,88].

As for the previously noted combined stressors, they have the worst effect in winter [9,16]. On top of the poor environmental conditions in winter and any potential parasite–host dynamic, the stress caused by cold is a real challenge for any parasite-threatened bee colonies [10]. With a growing environmental burden on honeybees, beekeeping techniques need to include strategies to help bee and colony resilience [4,39,89,90]. Understanding the consequences of all the factors cited above should help develop strategies to support the honeybees and develop better nourishment solutions for winter [16].

Knowing that immunity, nutrition, the microbiota, winter protection, mite parasitism, and virus transmission are part of this complex equation dealing with possible physiopathology, these factors must be taken into account in view of trying to maintain honeybee colonies. For instance, there are possible connections between immune pathways of the stress response to viral and mite attacks and the stress response to cold winters [1,11,16]. Another example is the role of glucose metabolism. Insects use glucose to initiate flight. However, for long-term flights (or other metabolic energy use), they require other types of fuels such as amino acids (proline) or fatty acids [12]. It is important to note that these kinds of important nutrients (i.e., amino acids and fatty acids) are not present in the commercially available food sold for bee wintering. In addition, some fatty acids provided by pollen nutrients inhibit pathogen bacteria sporulation in the brood [13]. Fatty acids are not supplemented in commercial feed even though it has been demonstrated that the omega-3 fatty acids are essential for learning and memory in honeybees [14,15,16].

Growing knowledge on the importance of nutritional resources in the environment and for bee wintering nutrient supplies bring the researchers to use the term of “Precision Nutrition”. Scientists study bee nutrition and nutrigenomics and actively evaluate key molecules that could be beneficial for better bee colony resilience facing the combined stressors of changing environments [1,17]. More specific nutritional supplements are evaluated [8,18,21,91,92,93,94]. We may not rule out this knowledge as part of possible future interventions and advice for beekeepers in view of further supporting the health of honeybees, as it has been done for human health in the past few decades. In the following part of this review, we discuss the current literature supporting the putative interests of using pollen, oils, phytonutrients, or other nutrients to support bee colony resilience.

### 3.1. What Would Be the Interest of Feeding Bees with Pollen?

As stated previously, some amino acids and lipids are key for the physiology of honeybees. Pollen is the main source of protein, amino acids, and lipids for honeybees along with the phytonutrients from nectar and resins [8,25,26,27,55,91,94,95,96,97,98,99]. Pollen is essential to the physiological development of adult bees and reduces their sensitivity to pathogens and pesticides [3,18,19]. Understanding bee physiology and their specific needs to thrive is essential to understanding why pollen is so important. The emerging bee will have to successively take on several roles divided between “in-hive” and “out-of-hive” duties [16]. At approximately one week of age, the young bees will nourish the brood, the emerging bees, and the queen. These nurses produce secretions, known as royal jelly, from their hypopharyngeal glands (HG) and mandibular glands (MG) [16,44,100]. HG are paired structures in the head producing the major protein fraction of the royal jelly. They exist throughout the Hymenoptera but become more developed and more productive in social insects.

HG are small in freshly emerged bees and reach maximum size and function within 8 to 10 days in the hive worker, which may be reduced to 3 days under specific environment contexts or colony needs [16,44,100]. The HG will degrade as the bees switch over to foraging activities. The normal age at first forage may vary, but usually occurs around 14 days of age. The typical nurse-to-forager transition is accelerated during stress [44,101].

In a stressed colony, bees might start foraging as early as 4 or 7 days of age. Stressed bees seem to have smaller HG and depleted abdominal lipids: octopamine-treated bees have smaller HG [16,102]. Wintering bees must have good lipid storage in the abdomen to survive. Experimental reduction of abdominal lipids induces precocious foraging, suggesting that abdominal lipid tissue might play a role in this behavioral transition. Foraging too early on has a negative impact on the individual and the colony’s health [16]. The forager’s searching behavior is compromised, and colony failures increase when foragers leave earlier than normal. HG are very sensitive to the presence of pollen in the hive [16,102].

Octopamine, an essential hormone regulating bee behavior, varies considerably with stress and pollen supply [103]. This hormone plays a crucial role in coordinating the metabolic, physiologic, and behavioral response of bees to modifications to their environment [16,103]. In a context of stress, octopamine mobilizes the stocked nutrients allowing the organism to access the necessary energy in its response to environmental challenges, accelerates the transformation of nurses to foragers, causes HG atrophy, and reduces abdominal lipids.

Nutrigenomics may help research on the interaction between food and metabolic pathways thus permitting analysis on how pollen nutrients might help honeybee health. The insulin/mTOR signaling pathway is a nutrient-sensing pathway linking food intake to animal growth, metabolism, reproduction, and lifespan. The genes from insulin/mTOR signaling pathways are upregulated by pollen nutrition in healthy bees. In honeybees, this pathway plays an important role in the regulation of the division of labor within the colony, and in aging. ILP-2, insulin-like peptide 2, is increased by pollen nutrition [3,20,21]. Amino acids in pollen seem to promote ILP-2 and might promote nursing activity and extend longevity. The opposite is observed with ILP-1 which is expressed at higher levels when nutrition is scarce. While pollen nutrition enhances macromolecule metabolism and activates the mTOR pathway required for tissue growth and development, it also contains nutrients that stimulate genes of longevity, such as genes coding for hormones and antioxidants (vitellogenin or superoxide dismutase) [8]. The negative impact of varroa mites on bee metabolism and immune function is not reversed by pollen feeding. Varroa parasitism prevents bees from receiving the beneficial effect of pollen. Mite infestation is extremely virulent by being linked to the viruses transmitted by the parasite.

Vitellogenin is a yolk protein taken up by the eggs and is associated with egg production by the queen [16,104]. Vitellogenin has an antioxidant function protecting bees against oxidative stress. Vitellogenin is considered as an indicator of longevity [8,57,104,105]. Pollen promotes the development of body fat and, via this effect, promotes vitellogenin production and longevity all together with the AMP (antimicrobial peptides), such as defensins and apidaecin. Varroa decreases vitellogenin production, and alters body fat and metabolism. Another analyzed gene, the Malvolio gene, codes for a manganese transmembrane involved in sucrose responsiveness. This gene is more active in forager bees, living more on sucrose than the nursing bees taking care of the brood inside the hive. Bees fed with sugar alone have a higher level of malvolio (*mvl*) expression in the abdomen. Normally, old foragers feed only on carbohydrates and young bees are fed with both pollen and carbohydrates. Young bees fed only with carbohydrates tend to have a similar gene expression to foragers [8]. As opposed to a poor diet, pollen is important for protein supply and for promoting the development of HG so important to nursing tasks. Varroa mite parasitism stimulates malvolio expression and inhibits some genes involved in insect immunity such as prophenoloxidase expression, consistent with the immunosuppression reported in parasitized bees. An interesting study exploring bee nutrigenomics shows that when comparing bees fed with sugar and pollen with those fed only on sugar, pollen will activate metabolic pathways helping bees to thrive and to improve nutrient-detection sensitivity [8]. Moreover, these nutrients from pollen had a positive effect on genes related to longevity, and on the production of antimicrobial peptides for the defense against pathogens (enhancing the Toll pathway spaetzle) [3].

Pollen alone is not sufficient but seems to be a necessary condition for bee resilience, thriving, and survival. Nectar from flowers is also considered an interesting fuel, but pollen is essential for organ development, and neurological and hormonal balances [3,22]. The accessibility of quality and diversified nutrients affects the development and the function of hypopharyngeal glands. The protein and lipid compounds of pollen play a major role in vitality and longevity of bees, having a major impact on their gut microbiota. Pollen scarcity causes the atrophy of glands vital for breeding, and reduces body weight, lipid and protein body composition, and bee longevity. Honeybees need pollen sources as soon as the queen lays her eggs at the end of wintertime [23]. Moreover, a study shows that protein- and lipid-metabolic pathways promote detoxification within bees, such as pesticide detoxification, and this all year round [24,25,26]. Pollen, a source of amino acids such as arginine, is indispensable for NO (nitrogen monoxide) synthesis in bees. NO is essential for the innate immune system, and activates antiparasitic systems and tissue repair. NO is part of the shared pathways of resistance to cold and viruses. Thus, pollen is considered as an important bee metabolic modulator, a source of lipids and proteins, and a hormonal and behavioral modulator of the colony.

### 3.2. Should We Add Essential Oils and Specific Phytonutrients?

**Figure 3:**
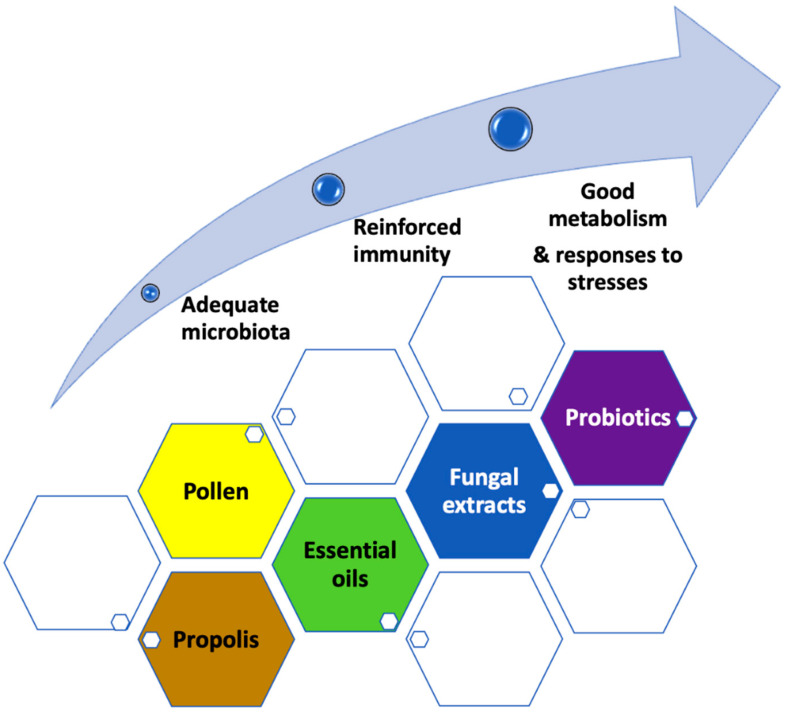
Bee nutritional approaches for reinforcing immunity and optimizing metabolism and responses to stresses.

Studies on phytonutrients and essential oils have shown an impact on maintaining bee metabolism and immunity, via either curative or preventive effect toward resistance for bee-colony pathogens (Table 1). Within bacteria, the two major pathogens are *Paenibacillus larvae* (American foulbrood) and *Melissococcus plutonius* (European foulbrood). The most aggressive viruses (DWV Deformed Winged Virus and ABPV Acute Bee Paralysis Virus) are those transmitted by mites. Fungi are also involved (*Ascosphera apis*) as intracellular parasites (*Nosema ceranae* and *Nosema apis* affecting gut epithelium) (Figure 2 and Figure 3) [14,106,107]. Among the different menaces, *Varroa destructor* (an external mite parasite) is thought to be one of the most important. Since the beginning of their evolution in their natural environment, bees have always been collecting a great diversity of nutrients produced by plants that ensure the balance of their metabolism, immune system, reproduction, and circannual rhythmicity with changing seasons depending on the geography and climate [12].

Over the last two decades, numerous studies have been performed to demonstrate the essential role played by different molecules found in plants, as carbohydrates in nectars, proteins and fatty acids of pollen, polyphenols or flavonoids from nectars, plant resins and pollen (Figure 3). The aim of these studies was also to provide scientific evidence that using them as food supplements would provide the hives specific supports, especially when the environment in impoverished, and when the honey has been harvested by humans for human needs [17,27,28,29,30,31]. Today, it is widely accepted that essential oils and phytonutrients (polyphenols, flavonoids, fatty acids, and proteins from pollen) have antimicrobial effects, they can enhance bee longevity, and can assist with an important reduction of *Nosema* proliferation. Those effects have been observed with plant extracts such as essential oils from mint, lemon balm, coriander, *Thymus vulgaris* or propolis, and clove oil, or with plant extracts such as caffein, kaempferol, gallic acid, coumarinic acid, quercetin, and garlic [32,33,34]. Amongst the essential oils active on *Penicillium larvae*, that from *Thymus vulgaris* is one of the most efficient [30,35,39,91,96,108,109,110,111,112,113,123,124,125,126].

### 3.3. Should We Add Propolis?

Honeybees collect antimicrobial plant resins in their environment and display them in the honeycomb in the form of propolis. The presence of propolis in the hive offers many advantages, especially in the resistance against pathogens. The botanical origin of propolis seems to be important.

Some molecules (flavonoids) of propolis could be particularly efficient against *Paenibacillus larvae*, an agent of the American foulbrood, and against *Ascosphera apis*, an agent of chalkbrood disease. The phenolic compounds derived from poplar resins would be quite strong inhibitors as the 3-acyl-dihydroflavonols extracted from *Populus fremontii* (Californian black poplar) [36,37,38,39,40].

### 3.4. Should We Add Fungal Extracts?

Highly infectious viral epidemics such as those transmitted by varroa have contributed to the recent severe decline of global honeybee health. Bees have been noticed harvesting on mushroom crops—those fungi known for their production of many chemical compounds with antibacterial, antiviral, or antifungal effects [121]. Mycelial extracts of several species of *Polyporus* known for their antimicrobial properties have been tested. Extracts from *Fomes* (*amadou*) and *Reishi* (*Ganoderma*) reduce the level of viral infestations such as the DWV (Deformed Wing Virus) and LSV (Lake Sinai Virus) correlated with the administered dose. While trials were performed in the beehive, colonies supplemented with extracts of *Ganoderma resinaceum* had a reduction of 79 times the count of DWV and 45000 times for LSV compared with the non-treated colonies. These results suggests that honeybees may benefit from these fungi and their antimicrobial compounds [41].

### 3.5. Therapeutic or Preventive Purpose

As shown in the literature, there are now more and more data supporting the potential use of different nutritional approaches for therapeutic or preventive purpose. However, we still lack knowledge about the precise doses useful to treat a diseased colony and about approaches for prevention, such as those found by the bees in their natural phytonutrients. Nevertheless, many efforts are being made in that direction [28,29,42,43]. Moreover, the composition of essential oils varies with the plant type, the soil, cultivation conditions, geography, and climate; there may also be great variations in chemistry (Table 1). Nevertheless, developing more complex food complements for wintering beehives might be considered.

Some bee food supplements have been evaluated considering colony growth, reduction of *Nosema* spores, mite infestation, viral load, and immune response [7,10,39,127,128].

## 4. Bee Microbiota Part of the Strategy for Bee Colony Survival

Expanding research on bee microbiota is unravelling the complex interaction between bees and microbes. Bees seemed to have appeared on the Earth 130 million years ago and the science of evolution says that the social organization of honeybees appeared approximately 80 million years ago. Ever since then, foragers have been collecting from plants for their specific nutrients which have evolved with specific phytonutrients, and have therefore developed a highly specific intestinal microbiota that only exists in their gut nowadays. The only way an emerging bee can get its indispensable gut microorganisms is by exchange with adult bees within the hive, and this has occurred over millions of years [44,45,46,47,48,49]. However, as discussed earlier in this review, the high colony losses are likely partly due to modified environments, including the introduction of pesticides, antibiotics, a poor environment with intensive monocultures of our modern agroindustry, and pathogens and parasites that are intensively exchanged with modern travel and export–import habits. Obviously, antibiotics used in modern beekeeping might play a role in reducing bee resilience [7,32,33]. Gut microbial balance is a major cornerstone in bee colonies’ resistance to environmental stressors. Gut symbionts influence bee immunity, resistance to pathogens, neuroendocrine pathways, food digestion and assimilation, growth, and longevity. One of the strategies to protect honeybee colonies could be to protect their gut microbiota by understanding how it thrives and what damages the delicate balance. In the article “missing microbes and bees” [24], the authors explain that for bees, as for humans, when microbial homeostasis is in default, it will impair immunity and hasten modern diseases.

A depletion of key symbionts impairs healthy physiological functions [52,53]. Worldwide studies are ongoing to understand what exact roles gut communities play on animal health. How much interacting or individual communities of symbionts impact the overall metabolic output of the host and of the whole microbial metabolism within the gut is still unknown (Interactomics–Metabolomics, etc. [54,55,56]). Honeybees are studied as models for microbiota research. Honeybee symbionts are relatively simple compared with other animal groups, as they are highly preserved and specific, and have very similar importance on host health [57,58]. Modern techniques available to study gut symbionts, such as DNA sequencing metagenomics, interactomics, and metabolomics, have allowed us to understand more of their impact on metabolism and functions within the microbial community and how they impact animal health. Core gut symbionts in bees are gut restricted, highly specific, and absent outside adult guts. They include both Gram-negative groups, such as very closely interacting *Gilliamella apicola* and *Snodgrassella alvi*, and Gram-positive groups such as *Lactobacillus species* and *Bifidobacterium species*. Approximately eight bacterial species are making 95% of the gut bacteria in most *Apis mellifera* individual workers. Each cluster corresponds to closely related bacterial strains. A 2020 consensus accepts that this core microbiota consists of 6 to 10 phylotypes consistently found in honeybees, independent of geography [52]. Three species are within the Gram-negative phylum Proteobacteria: *Snodgrassella alvi*, *Gilliamella apicola*, *Frischella perrara*. They are restricted to *Apis* species and *Bombus*, and *Frischella perrara* is restricted to honeybees. Three closely related clusters of Gram-positive bacteria are also restricted to bee guts. The Firmicutes phylum is characterized by *Lactobacillus firm 4* (or *L. mellis*, *L. mellifer* strains) and *Lactobacillus firm 5* (or *L. helsingborgensis*, *L. melliventris*, *L. kimbladii* strains). The Actinobacteria phylum is characterized by a *Bifidobacterium* cluster (*B. asteroides*, *B. actinocoloniiforme*, *B. bohemicum* strains). Two other species clusters are related to Alphaproteobacteria: *Alpha 1*, which is similar to the *Bartonella species*, and *Alpha 2* (*Alpha 2*-*1*, which is a gut specialist, and *Alpha 2-2 Parasaccharibacter apium* that commonly grows outside in the environment and is present in the larval gut, adult crop, nectar, honey, and hive materials, but is absent from the adult hindgut). *Lactobacillus kunkei* has been found in the hive, larval gut, adult crop, honey, and nectar, but not in the adult hindgut [11,23,24,60,62,63,67,69,70,71,72,76,77,79,82,83,87,129,130,131]. Some other clusters of Bacteroidetes may be present in low abundance, or Enterobacteriaceae may be present with common insect pathogens [59,60]. The colonization of newly emerged bees goes through a fecal–oral route. The bees must be exposed to the hindgut contents of the nurses in the hive. Therefore, large spectrum antibiotic treatments in the hive can be deleterious for the essential microorganisms involved in bee metabolism. The mature worker bee has approximately 1 billion bacterial cells, 95% of them in the hindgut. The crop (stomach) contains more of the environment *Lactobacillus* (*L. kunkei*) and the nonspecific *Alpha 2-2* (*Parasaccharibacter*). The midgut contains very few bacteria, which are more concentrated towards the hindgut. The hindgut has two compartments: the ileum (a very narrow space with six longitudinal invaginations) and the rectum (a sac-like space). Each region has very specific bacteria with highly specific tasks. The ileum microbiota is dominated by the Gram-negative species, as *Gilliamella apicola* and *Snodgrassella alvi* which form a dense biofilm beginning at the junction of the Malpighian tubes all along the ileum tube. It contains also *Frischella perrara* which is very restricted to the pylorus and at the beginning of the ileum, and which dominates at the nurse stage (8 days of life). *Snodgrassella alvi* is right on the epithelial gut layer, and *Gilliamella apicola* is toward the lumen; these two strains of bacteria tightly interact and are interdependent. They are hyperspecialized in the digestion of carbohydrates and pollen-containing foods. There is a complementary metabolic activity between *G. apicola* which contains great numbers of glucose-metabolic pathways and *S. alvi* that cannot use glucose (but can use the carboxylates produced by the downstream sugar metabolism of *G. apicola*). The metabolites produced by *S. alvi* are essential for gut epithelium trophicity, the production of antimicrobial proteins, and vitellogenin synthesis (essential for bee longevity). This bacterial community has co-evolved with the honeybee adult gut and is its sole ecological niche.

The rectum is the richest part of the gut microbiota and is dominated by *Lactobacillus* and *Bifidobacterium* clusters. They also play an essential role in carbohydrate catabolism, and in the nutrition of their host. Besides the composition of the gut microbiota, the metabolic functions are also important. Recent studies have analyzed metabolic functions of bacteria in the honeybee gut [3,58,61,62,63,64]. The metabolic activity of key symbionts has important interactions that have marked effects on the host. Microbial communities may organize in food chains (i.e., cross-feeding), where one species produces metabolites useful to another and, finally, to the host. Honeybee microbiota utilizes a wide range of pollen-derived substances. Gut symbionts are important for: the digestion of non-digestible plant structures; pollen-derived structures; fermentation and production of organic acids and aromatic compounds important for host metabolism energy and physiology; and intestinal epithelial trophicity production of essential nutrients absent from host food such as amino acids. They also have a major role in retrieving host metabolic wastes, in the regulation of hormone production such as juvenile hormone or vitellogenin, and are important for growth, development and reproduction, the pacing of bee behavior within the hive, and for longevity. They have an impact on neurophysiology through the modulation of serotonin, octopamine, and dopamine. They favor the production of AMP and regulate the immune system. They play a crucial role in the detoxification of ingested toxins and in resistance against pathogens via effect on the immune system, AMP, and biomass effect [52,56,58,65,66,67]. Due to its close contact with gut epithelium, the microbiota conveys information to the host. Enteroendocrine cells respond to nutrients and digestive metabolites via G protein-coupled receptors leading to a release of neuropeptides such as Neuropeptide F or Allostatins, and via regulating food behavior, reproduction behavior, and hormone signaling. Enteroendocrine cells are also stimulated by organic acids produced by symbionts in the gut, derived from the fermentation of dietary fibers [59,88]. They have a profound and conserved effect on host metabolism [68,69]. Substrates of gut bacteria originate mostly from the diet, making diet a major modulator of gut symbiont composition and metabolism and having an important impact on host physiology and global health. Evidence is arising showing how the biodiversity of nutrients is important for animal health and, more specifically, for bee colony resilience to environmental challenges [3,8,58]. Studies using pollen restriction, organic acid feeding and nutrigenomics on honeybees show how the diet–microbiota–host axis could be part of the current, developed efforts to help colonies [3,61]. Modern challenges for the honeybee gut symbionts consist of poor environments where vast monocultures with no floral diversity invade large landscapes, seasonal changes, bee feeding with single sugar food supplements, pathogens and parasites from the varroa mite spreading viruses, antibiotics and drugs, air pollution, pesticides, microplastics (Figure 1 and Figure 2), and an ever-growing number of chemical pollutants added to the environment progressively disrupting the microbial balance.

There is a bidirectional regulation between the innate immune system and the microbiota. Honeybees rely on microbial constituents to resist against pests and pathogens, to detoxify toxins, and to overcome periods of limited food resources. Dysbiosis is defined as an alteration in microbial balance leading to a disruption in function in a site such as the gut. Mild dysbiosis is considered as a transient fluctuation in a core species in the midgut and ileum which can generate poor host development, early mortality, increased susceptibility to infection via breakdown of symbiotic film, and an alteration of host gut tissue. Severe dysbiosis affects the whole gut (with the rectum) with a drastic disruption of core microbiota, leading to opportunistic and larval pathogens, dysfunctional social behavior and, finally, colony collapse disorder [52,70,71,72]. Microbial solutions with *Lactobacillus* species have been showed to provide protection against pathogens, helping pesticide detoxification, and with *Bifidobacterium*, better egg production, better harvesting, nutrient promoting, and pathogen prevention [70,73,74]. Engineered *Snodgrassella alvi* helps *Apis mellifera* from mites and their viral load [75,76]. A recent article [77] shows that flowers may be hubs of microbial transmission and reflects a flower–bee interaction network. These microbial routes might obviously be more predominant for solitary bees; however, this could be interesting to look at for social bees as a meta-network dynamic. Health is a general weblike dimension englobing plants, animals, and humans [78]. The microbiota of the hive materials seems linked to variations of environment. Environmental floral diversity is essential for global health.

## 5. Conclusions

Bee health is linked to the complexity of multiple factors (Figure 1). It can be considered as a “Systems Medicine” (Figure 1). Published reports have increasingly recognized that effective solutions demand the collaborative approaches of many disciplines [3,12,132,133,134]. Understanding bee health comprises studying and combining genetics, nutrition, microbiology, animal and plant biology (insects as individual and social organization), environmental science, agronomy, toxicology, biochemistry, beekeeping techniques, etc. (Figure 1) [1,11,12,105,132,134,135]. Groups of researchers are emphasizing and supporting a collaboration between scientists, beekeepers, and farmers for future field experiments to allow for a better understanding of the complex biotic, abiotic, and social matrices shaping the health and disease of colonies [1,14,39,132]. Further research should integrate observation and the participation of beekeepers and farmers with scientists working on honeybee health (Figure 1). Projects to develop biological control (pheromones, endosymbionts, and entomopathogens) and to develop drugs, organic acids, or phytotherapeutic supplements to help overcome the parasite have been studied. Researchers and beekeepers are selecting naturally resistant colonies: VSH (varroa sensitive hygiene bees) [8,30,37,78,90,123,134,136,137,138]. In the article,” Reconnecting for our Future”, the lancet One Health Commission [1] explains working on several possible approaches to examining the “animal–environment–human” interface with three separate but interconnected dimensions. The first is how humans and animals share the same environment and with what consequences. The second is about safe food and food systems. The third is shared medicine and interventions. Knowledge and evidence generation should evolve in an integrated understanding of the complexity. Bee colonies have a major role in the system as major pollinators. They contribute to the food supply by pollinating crops; however, bee health is endangered by climate change, loss of biodiversity, burdens caused by pathogens, predators, and toxins.

Finally, the scientific community is progressively developing future strategies concerning bee health and supporting bee resilience. Given rapidly changing environments, and animal and human health systems, scientists should work synergistically to ensure a sustainable, reconnected approach of bee resilience.

## Figures and Tables

**Table 1:** Proposition for an example of potential bee feeding solution composition *.

Composition of the Solution	Quantities
A basic commercial product of fruit juice	For example: *HAPPYFLOR Z* (10 L) made from fruit juice, beet extract, chicory in variable proportions
Pollen	Ideally from your bees (250 g)
Honey	Ideal source from Our bees (2 kg)
Propolis	in alcoholic solution (5 mL)
Essential oils ^#^: *Rosmarinus officinalis* *Thymus vulgaris* *Laurel nobilis* *Eucalyptus globulus*	5 drops of each
Apple vinegar	5 tablespoons

* Personal communication from the authors based on the current literature and the literature describing the actions of the different compounds described. ^#^ All these essential oils may have different chemical variables depending on their origin, but they contain the chemical compounds described in the studies [7,30,31,39,68,91,93,96,108,109,110,111,112,113,114,115,116,117,118,119,120,121,122]: 1,8-cinéol (eucalyptol): *Laurel nobilis*, *Rosmarinus officinalis*, *Eucalyptus globulus*; alpha pinene: *Laurel nobilis*, *Rosmarinus officinalis*; alpha-caryophyllene: *Thymus vulgaris*, *Rosmarinus officinalis*; alpha-terpineol: *Laurel nobilis*, *Thymus vulgaris*; beta-phellandrene: *Eucalyptus*.

## Data Availability

Not applicable.
